# Tracking functional brain networks in preterm and term infants using precision mapping

**DOI:** 10.1016/j.dcn.2025.101629

**Published:** 2025-10-12

**Authors:** Diego Derman, Silvina L. Ferradal

**Affiliations:** Department of Intelligent Systems Engineering, Indiana University, Bloomington, IN, USA

**Keywords:** Premature birth, FMRI, Functional connectivity, Precision functional mapping, Individualization

## Abstract

Preterm birth is a known risk factor for neurodevelopmental disabilities, but early neurobehavioral assessments and structural imaging often fail to predict long-term outcomes. This limitation underscores the need for alternative biomarkers that reflect early brain organization. Resting-state functional connectivity offers a powerful tool to track functional brain organization by characterizing resting-state networks (RSNs), potentially offering more sensitive biomarkers. However, most fMRI studies in infant populations use group-level analyses that average subject-specific data across several weeks of development, reducing sensitivity to subtle, time-sensitive deviations from typical brain trajectories, particularly in higher-order association networks. Using a recently introduced precision mapping approach, we estimated individual resting-state networks (RSNs) in a large cohort of term and preterm neonates from the developing Human Connectome Project. RSN connectivity strength increased linearly with age at scan, with primary sensory networks maturing earlier and higher-order association networks, including the default mode network (DMN), showing more gradual but pronounced changes, evolving from an immature organization in preterm infants to a more adult-like pattern in term-born infants. Longitudinal data from a subset of preterm infants confirmed ongoing network development shortly after birth. Despite this maturation, preterm infants did not reach the connectivity levels of term-born infants by term-equivalent age. These findings demonstrate that individualized RSN mapping captures heterogeneous developmental trajectories in the neonatal brain and highlights higher-order association networks, particularly the DMN, as promising early markers for monitoring neurodevelopmental outcomes in neonates.

## 1 Introduction

Preterm birth is a well-established risk factor for neurodevelopmental disabilities later in life (Bhutta et al., 2002, Hadaya et al., 2025, Marlow et al., 2005, Saigal and Doyle, 2008). Yet, early neurobehavioral assessments and structural imaging often fail to predict which infants will experience long-term impairments, limiting opportunities for timely and personalized interventions (Spencer-Smith et al., 2015, Spittle et al., 2015). For example, developmental coordination disorder (DCD) and cerebral palsy (CP), two conditions associated with premature birth, are sometimes diagnosed in children with no detectable injury on structural magnetic resonance imaging (MRI) at birth (Spittle et al., 2021). Because DCD is typically not diagnosed until around age 5, the absence of early clinical signs represents a missed opportunity for intervention. Similarly, motor assessments at age 2 have limited predictive value for later motor outcomes (Luttikhuizen Dos Santos et al., 2013, Montgomery et al., 2023, Spittle et al., 2013) and show weak correlations with structural imaging.

Resting-state functional connectivity MRI (fcMRI) provides a powerful tool to track early brain development by characterizing resting-state networks (RSNs), potentially offering more sensitive biomarkers of atypical outcomes than behavioral or structural measures alone. Studies in term and preterm-born infants have shown that cortical maturation follows heterogeneous patterns, with sensory–motor and visual networks emerging early, while higher-order association networks, such as the default mode network (DMN), emerge more gradually (Cao et al., 2016, Doria et al., 2010, Eyre et al., 2021, Smyser et al., 2010, Sydnor et al., 2021, Sylvester et al., 2022). This developmental sequence, from primary sensory to association regions, likely contributes to greater inter-individual variability in association areas during early life. Nevertheless, most studies in infants have relied on group-level analyses, which may obscure meaningful individual differences (Gordon et al., 2022). Averaging across multiple infants can bias group-level functional topography toward the more consistent primary sensory regions, underrepresenting association networks. As a result, group-averaged maps may produce fragmented representations of association networks (e.g., isolated clusters of the DMN) and developmental trajectories in which statistically significant age effects are more frequently detected in primary sensory networks.

Addressing this limitation requires precision mapping approaches that preserve individual-level functional topography, enabling more accurate characterization of early network organization that could potentially be linked with later behavioral outcomes. However, mapping individual-level RSNs in neonates, particularly those born preterm, remains challenging, as the inherently low signal-to-noise ratio (SNR) of short-duration scans typical in neonatal imaging leads to noisy estimates using standard statistical approaches. In adults, robust individualized RSN mapping typically requires several hours of functional data (Gordon et al., 2017, Laumann et al., 2015), far beyond what is feasible in infants due to motion, cost, and tolerance.

In our previous work, we introduced a surface-based Bayesian framework to map individual-level RSNs in term-born neonates using short-duration fMRI scans (Derman et al., 2025). This approach increases the effective SNR by shrinking noisy regions toward a population-informed template while preserving meaningful individual variability. A key capability of this framework is the generation of statistically thresholded, RSN-specific masks for each subject, enabling individualized functional parcellations, an advance over standard neonatal pipelines that rely on group-level parcellations. By capturing subject-specific network topography under these practical constraints, this framework illustrates how precision mapping approaches can be applied to neonates to characterize developmental trajectories with greater specificity.

In this study, we extend the analysis to a cohort of > 350 preterm and term-born infants from the large, publicly available developing Human Connectome Project (dHCP) dataset (Edwards et al., 2022) to investigate the impact of prematurity on individualized functional network organization. This work represents, to our knowledge, the first large-scale application of advanced individualized functional mapping methods to premature infants.

We hypothesize that individual-level RSN mapping in term and preterm infants will reveal age-related effects in (1) individual functional topography as well as (2) developmental trajectories of both primary sensory and association networks. Furthermore, we hypothesize that the most pronounced maturational effects will be present in association networks such as the default mode network, which will evolve from an immature state to one that more closely resembles its adult organization. These findings could inform future studies by highlighting association networks as promising candidate biomarkers for tracking individual neurodevelopment in this population, ultimately aiding in the early identification of at-risk infants and the design of personalized interventions.

## 2 Methods

### 2.1 Subjects and MRI data acquisition

MRI data was obtained from the second release of the developing Human Connectome Project (dHCP) database. For this study, we included subjects with complete functional imaging sessions that passed quality control and had a radiological score of two or lower, indicating no lesions of clinical or analytical significance. Based on these criteria, 391 sessions corresponding to 371 subjects (age at birth: 25.6 – 42.3 weeks gestational age, GA) scanned shortly after birth or at term-equivalent age (age at scan: 29.3 – 44.9 weeks postmenstrual age, PMA) were considered for further analysis.

All scans were obtained with a 3 T Philips Achieva using a neonatal head coil at Evelina Newborn Imaging Centre, St. Thomas Hospital, London, UK. Both T1-weighted (TR = 4795 ms; TE = 8.7 ms) and T2-weighted (TR = 12 s; TE = 156 ms) structural scans were obtained with a multi-slice Turbo Spin Echo (TSE) sequence, with in-plane resolution 0.8 × 0.8 mm² and 1.6 mm slices overlapped by 0.8 mm. Two stacks of images were taken per weighting, sagittal and axial, which were integrated to obtain T2w volumes with an image resolution of 0.8 mm isotropic. Blood oxygen level-dependent (BOLD) scans were obtained with a multi-slice gradient-echo planar imaging (EPI) sequence (TE = 38 ms; TR = 392 ms, multiband factor = 9; flip angle = 34°) with an image resolution of 2.15 mm isotropic. A resting-state BOLD fMRI acquisition of 2300 time points (15 min) was obtained for each infant.

### 2.2 MRI Preprocessing

Structural and functional MRI data were preprocessed as described in Derman et al., (2025). Briefly, individual cortical surfaces from the dHCP minimal processing pipeline (Makropoulos et al., 2018) were aligned to a 40-week symmetrical atlas (Williams et al., 2023) using the Multimodal Surface Matching tool based on cortical folding (Robinson et al., 2018). Resting-state BOLD fMRI data from the preprocessed dHCP pipeline (Fitzgibbon et al., 2020) were projected onto each individual surface using a version of the HCP surface pipeline (Glasser et al., 2013) modified to suit the neonatal population, masking out the subcortical voxels, and subsequently aligned to the cohort-specific 40-week atlas. Geodesic 2D Gaussian smoothing was applied using a 3 mm FWHM kernel.

Following a conservative frame censoring approach, a contiguous block of 1600 frames (10 min) with the minimum number of motion outliers (as measured by DVARS) was retained for each subject. Frames with DVARS higher than 1.5 times the interquartile range above the 75th percentile within a session were considered corrupted by motion. Sessions with more than 160 motion-corrupted volumes (10 %) were excluded. Based on this criterion, 369 sessions from 352 subjects (274 term-born and 78 preterm infants) were included in the analysis. Table 1 presents a summary of the demographics considered in the analysis.

**Table 1:** **Study population.** After excluding subjects with excessive motion artifacts, 352 infants with 369 scans from the second release of the dHCP dataset were included in the present study. Participants were categorized into three groups - preterm, term-equivalent, and term - based on their birth age and age at scan.

	**Preterm**^a^	**Term-equivalent**^b^	**Term**^c^
**Subjects**	52#	43#	274
Birth age (weeks)*	32.8 ± 2.7 (25.6–36.4)	32.5 ± 3.1 (27.1–36.9)	40 ± 1.2 (37.1–42.3)
Age at scan (weeks)*	34.7 ± 1.7 (29.3 – 36.9)	41.4 ± 1.9 (37 – 44.9)	41.2 ± 1.7 (37.4–44.9)
**Female/Male**	15/37	15/11	121/153

* Mean ± Standard Deviation (Min. - Max.)

# Note that the “preterm” and “term-equivalent” groups do not completely overlap. Only 17 infants were scanned shortly after premature birth and again at term-equivalent age.

a Born and scanned before 37 weeks of postmenstrual age (PMA).

b Born before 37 weeks PMA but scanned at or after 37 weeks PMA.

c Born at or after 37 weeks PMA.

### 2.3 Subject-level estimation of RSN maps

To obtain individual resting-state network (RSN) maps, we used a Bayesian hierarchical model that incorporates population information through empirical priors (Mejia et al., 2020). For this study, the empirical priors include estimates of the mean and between-subject variance of RSNs from a representative sample of 36 infants uniformly distributed across the entire cohort (age at scan: 36.6 – 44.9 weeks PMA, birth age: 29.4 – 41.9 weeks PMA). Note that the subjects included in the empirical priors were removed from further analysis.

To construct these priors, we first performed group independent component analysis (ICA) on a subset of 24 infants (age at scan: 44.5 – 45.5 weeks PMA). Based on visual inspection of the group ICA maps, associated time series, and power spectra, we identified eight RSN maps: lateral motor, medial motor, somatosensory, auditory, primary visual, motor association, visual association, and default mode network. We then applied dual regression to the 36-subject representative sample to obtain rough estimates of individual RSN maps, which were then used to compute the mean and the between-subject variance of each of the eight RSNs.

Using the empirical priors along with subject-specific fMRI data as inputs to an expectation–maximization (EM) algorithm, we obtained subject-level estimates of the posterior mean and standard deviation for each RSN map. From these estimates, subject-specific t-statistic maps were computed. The posterior distribution maps are highly robust to noise variance (Mejia et al., 2018) and enable formal inference through null-hypothesis testing to derive subject-specific binary masks of statistically significant engagement for each RSN map. Finally, the t-statistic values were averaged within the subject-specific binary mask to obtain a more accurate metric of RSN strength. For a detailed description of the Bayesian framework, see our methodological paper (Derman et al., 2025). The in-house code used includes shell scripts, python scripts, and R implementations of the templateICAr (https://github.com/mandymejia/templateICAr) library, a version of the code, edited for clarity, is available and maintained at https://github.com/FerradalLab/babyBayes.

## 3 Results

**Fig. 1:**
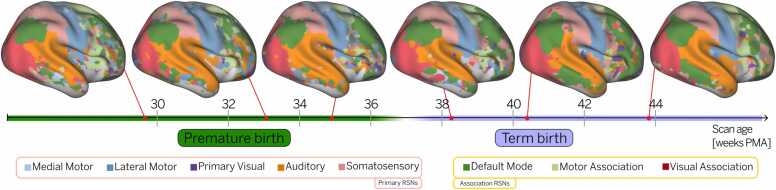
Individual-level parcellations reveal system-level differences in preterm and term-born infants. Functional parcellations for six randomly selected subjects scanned at different ages. The position of each subject on the timeline reflects their postmenstrual age (PMA) at the time of scanning (29.9, 32.6, 35.1, 38.1, 40.1, and 43.9 weeks PMA, respectively). Subjects born at or after 37 weeks PMA are considered term-born. Functional parcellations were obtained using a winner-takes-all (WTA) approach, where each vertex was assigned to the resting-state network (RSN) with the highest value at that location. The results were projected onto a 40-week cortical surface atlas for visualization purposes.

Individual-level functional parcellations reveal system-level differences between preterm and term infants, as illustrated by representative subjects from the full cohort (Fig. 1). Note the stability of the primary RSNs, such as medial and lateral motor networks. In contrast, higher-order RSNs seem to be still developing, as evidenced by the prefrontal and temporal clusters of the default mode network (DMN) and the motor association network.

### 3.1 Longitudinal trajectories of RSNs in premature infants

**Fig. 2:**
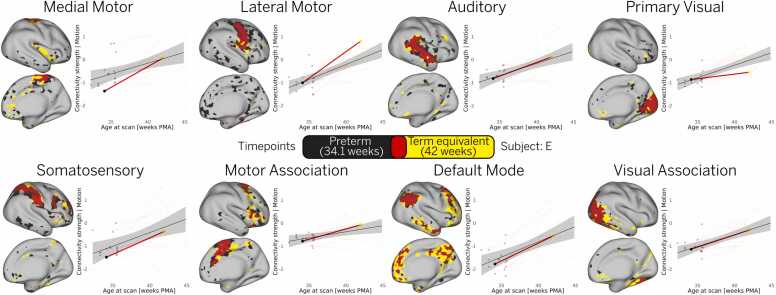
Longitudinal trajectories in premature infants show increased connectivity strength across networks. The relationship between age at scan and individual connectivity strength was examined in 15 preterm infants, each scanned twice: once at preterm age and again at term-equivalent age. The solid black line represents the group-level linear trend, with the shaded gray area indicating the 95 % confidence interval. For illustration, we highlight one representative infant from this cohort, showing the individual linear trend (red) across the two scanning sessions, along with the associated functional parcellation maps for each RSN. In these maps, black, yellow, and red regions indicate parcels present at the first session (34.1 weeks PMA), the second session (42 weeks PMA), and their spatial overlap, respectively.

To characterize the maturation of RSNs in premature infants, we analyzed longitudinal changes in connectivity strength in a cohort of 15 infants scanned at two time points: once shortly after birth during the preterm period, and again at term-equivalent age. Note that two preterm infants from this longitudinal sample were excluded from further analysis because their data were used to construct the empirical priors. To quantify these changes, we first generated binary masks for each individual RSN by thresholding the t-statistic maps to identify regions of significant activation (see Methods). Then, we calculated mean connectivity strength for each individual RSN as the average t-statistic value within each mask, weighted by the surface area of each vertex. To minimize potential bias from motion differences between scans, we regressed out motion effects using a linear model. The model confirmed no significant differences in motion between the two sessions. Finally, we used a linear regression model to estimate the average maturation between the first and second scans. Across time, we observed increases in functional connectivity strength in all RSNs (Fig. 2), consistent with ongoing network maturation during this critical developmental window.

We also observed topographical changes in some RSNs between the two scans, as illustrated by individual-level functional parcellation maps from a representative infant in this cohort (Fig. 2). Notably, the primary RSNs remained relatively stable, while topographical expansion was observed in the association RSNs, particularly the DMN.

### 3.2 Trajectories of RSNs at term-equivalent age

**Fig. 3:**
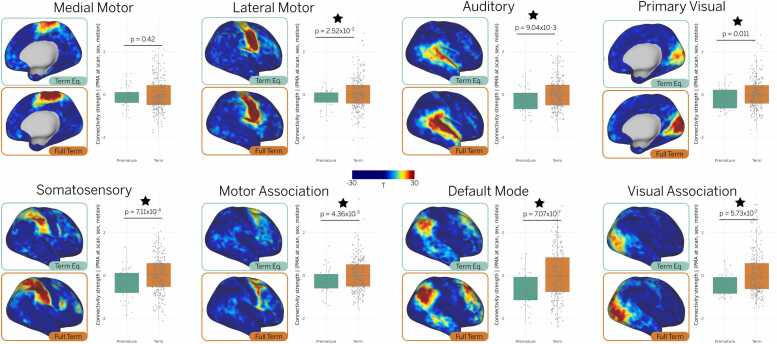
Functional connectivity profiles show differences between preterm and term-born infants at term-equivalent age. Group-average t-statistic maps for term-born (N = 247) and preterm infants scanned at term-equivalent age (N = 37) reveal generally weaker RSNs in preterm infants. This finding is supported by *t*-test comparisons, which show significantly lower connectivity strength in preterm infants across all networks except the medial motor network. For visualization, group maps are projected onto the 40-week inflated atlas. Stars represent statistically significant differences between groups (p < 0.05).

Next, we examined whether the developmental trajectories of preterm infants converged with those of term-born infants by term-equivalent age. To investigate this, we compared the spatial topography and connectivity strength across all RSNs between preterm infants scanned at term-equivalent age (N = 37; 37–44.9 weeks PMA) and term-born infants scanned within the same age range (N = 247). Note that six preterm infants from the term-equivalent group and 27 from the term-born group were excluded from further analysis because their data were used to construct the empirical priors. Qualitative comparisons of group-average t-statistic maps revealed weaker connectivity patterns across all networks in preterm infants at term-equivalent age compared to term-born infants. Furthermore, a two-sided *t*-test of connectivity strength, adjusted for sex, motion, and scan age, revealed statistically significant differences across all networks except the medial motor network (Fig. 3).

### 3.3 Effect of age and prematurity

**Fig. 4:**
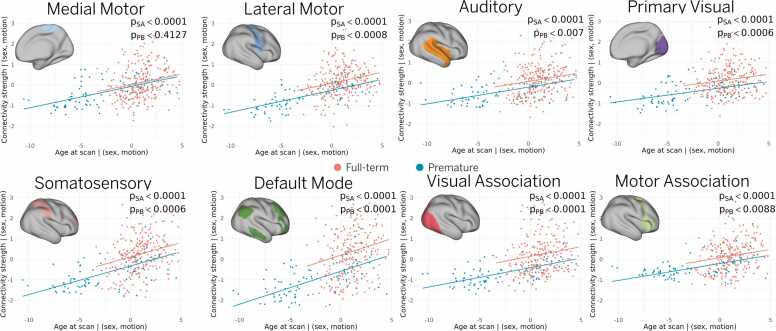
Effect of age and prematurity. A linear regression model was used to assess the relationship between individual connectivity strength and scan age across the entire cohort, controlling for sex and motion. All RSNs exhibited significant age-related increases in connectivity strength. However, preterm infants showed significantly lower connectivity than term-born infants in every network except the medial motor (as denoted by p_PB_). This offset persisted across the studied age range, with no difference in maturation rate between preterm and term-born groups. Note that p_SA_ refers to the p-value for the linear term in the regression model, which captures the association between connectivity strength and age at scan (i.e., maturation). A value below 0.05 indicates a significant positive association in term-born infants, suggesting that connectivity increases with age. Similarly, p_PB_ refers to the p-value for the categorical variable “preterm birth.” A value below 0.05 indicates that preterm infants exhibit significantly lower connectivity strength compared to term-born infants. For all RSNs, the p-values for the interaction term between “preterm birth” and “age at scan” were greater than 0.05, indicating that the relationship between connectivity and age does not significantly differ between term and preterm infants (i.e., both groups show a similar maturation pattern over time).

Finally, we aimed to characterize the entire developmental trajectory of RSNs in term- and preterm-born infants during the early postnatal period. For each session in the cohort, we computed connectivity strength from individual-level RSN maps estimated with our Bayesian model and fit a linear regression with connectivity strength as a function of scan age. The regression model controlled for sex and motion and included an interaction term for prematurity to assess its influence on RSN maturation. A statistically significant relationship between connectivity strength and age at scan was found for all RSNs (Fig. 4). Furthermore, preterm infants show a significantly lower connectivity strength than term-born infants for all networks except medial motor. No significant interaction effect between prematurity and age at scan was observed, confirming that the developmental trajectories of preterm infants do not converge with those of the term-born infants within the studied age range.

## 4 Discussion

This study advances our understanding of how premature birth affects functional brain development by introducing a precision mapping approach. In this study, we used the term “precision mapping” to emphasize individualized estimation of RSN topography under the constraints of neonatal imaging, rather than implying the extended acquisition times often required in adults. This framing highlights how individualized approaches can still yield reliable and developmentally meaningful insights, even with short-duration scans. Consistent with prior research, we observed overall weaker functional connectivity in preterm infants at term-equivalent age compared to term-born neonates (Eyre et al., 2021, Smyser et al., 2010, Sun et al., 2024). Our results also capture the ongoing maturation of resting-state networks (RSNs) during the early postnatal period, with significant increases in functional connectivity strength observed across all cortical networks.

### 4.1 Individual-level precision mapping of functional maturation

A central contribution of this work is the ability to examine individual-level RSN topography in preterm and term-born neonates, enabling a more detailed characterization of the heterogeneous patterns of cortical development. This was made possible by a precision functional mapping approach (Derman et al., 2025), which combines surface-based alignment, age-matched atlases, and Bayesian modeling to produce cleaner subject-level RSN estimates. These methodological advances allowed us to test our hypothesis that individualized mapping would better capture higher-order association networks, which are typically underrepresented in group-level analyses due to their greater inter-individual variability. As a result, we were able to track topographical changes in individual RSNs at different ages (Fig. 1), revealing patterns that traditional group-level approaches cannot detect. Our results suggest that higher-order RSNs undergo accelerated maturation following premature birth (Fig. 2) and show the largest differences in connectivity strength between preterm and term-born infants at term-equivalent age (Fig. 3).

Among all networks, the default mode network (DMN) exhibited the most pronounced developmental trajectory, with the largest difference between preterm and term-born infants (Fig. 3) and the steepest slope in maturation rate (Fig. 4). This rapid growth was also reflected in topographical changes, particularly in the frontal and temporal clusters, in both cross-sectional samples (Fig. 1) and a smaller longitudinal sample (Fig. 2). While previous studies reported postnatal development of the DMN (Doria et al., 2010, Sylvester et al., 2022), our findings reveal more complete interconnection between DMN clusters than previously observed (Eyre et al., 2021, Myers et al., 2024, Smyser et al., 2010), highlighting the benefit of individualized mapping for capturing higher-order association network topography.

Functional brain organization is well established and shaped by the prenatal environment during the third trimester of gestation (Kostović et al., 2019, Scheinost et al., 2024, Van Den Heuvel et al., 2015). Recent studies suggest that differences in functional connectivity between infants who will be born preterm and those who reach full term may already be detectable in utero (Thomason et al., 2017, Van Den Heuvel and Thomason, 2016). In this context, the absence of a significant difference in the slopes between the term and preterm developmental trajectories observed in this study (Fig. 4) may indicate that functional connectivity differences observed at birth persist through the neonatal period studied here, without evidence of convergence. Nevertheless, more comprehensive fetal-neonatal longitudinal studies are required to enable direct comparisons across cohorts and developmental stages.

### 4.2 Implications for early biomarkers

Precision functional mapping techniques have traditionally relied on large volumes of individual fMRI data, often collected over several hours (Gordon et al., 2017). Here, we demonstrate that surface-based Bayesian modelling allows enhanced estimation of individual RSNs from short-duration neonatal scans. This capability is particularly important for higher-order association networks, which exhibit more heterogeneous developmental trajectories and inter-individual variability. When combined with other biomarkers collected later on, this approach could support the early identification of atypical developmental trajectories and inform personalized interventions.

Building on this, our subject-level analyses in the preterm cohort revealed both ongoing maturation and a significant reduction in functional connectivity strength across RSNs. These findings, in combination with recent studies linking structural MRI abnormalities (Pagnozzi et al., 2025, Trimarco et al., 2024) and diminished functional connectivity (Cyr et al., 2025) to motor outcomes in preterm infants, underscore the potential of individualized RSN maps as valuable tools for assessing early brain development and the potential impact of prematurity and other early-life exposures.

Given its rapid postnatal maturation and the pronounced differences observed between term and preterm infants, the DMN is a strong candidate biomarker for tracking early brain development. The fact that individual differences in the DMN are greater than in primary sensory networks, likely reflecting its later developmental trajectory, may also indicate greater inter-subject variability (as seen in the interquartile range, Fig. 3). This makes it especially promising for studying behavioral outcomes in early life, particularly as differences in DMN connectivity have already been linked to neurodevelopmental disorders like autism spectrum disorder (ASD) (Haghighat et al., 2022).

Although we did not observe significant associations between RSN strength at birth and Bayley scores at age 2 in this cohort, this finding aligns with prior evidence that early Bayley assessments, even at later ages, provide limited predictive power for long-term neurodevelopmental outcomes (Cyr et al., 2025, Luttikhuizen Dos Santos et al., 2013, Montgomery et al., 2023, Spittle et al., 2015). In this sample, neither language nor cognitive Bayley scores differed between preterm and term-born infants, further highlighting the limited sensitivity of these measures at this developmental stage. Notably, our analyses were restricted to cortical networks, whereas recent studies indicate that thalamo-cortical and subcortical functional connectivity at birth may show stronger relationships with later developmental outcomes (Canini et al., 2025, Toulmin et al., 2021). Future work extending our Bayesian framework to include subcortical regions and leveraging longer-term follow-up datasets, such as the HEALthy Brain and Child Development (HBCD) study (Dean et al., 2024), may provide more robust insights into the predictive value of early functional connectivity.

### 4.3 Methodological considerations

Although the longitudinal sample included only 15 infants, the data provide valuable insights into individual developmental trajectories. Larger longitudinal cohorts will be critical to better capture inter-individual variability and refine developmental trajectory estimates. Direct comparison of longitudinal trajectories between preterm and term-born infants would also complement our cross-sectional findings (Fig. 4) and yield a more complete picture of cohort differences.

The current sample was also not ideally suited to examine the effects of gestational age as a continuous variable. In particular, the birth ages of subjects with term-equivalent scans (Fig. 3) were not evenly distributed, limiting our ability to assess the dose-dependent effects of prematurity. This remains an important direction for future research, as prior studies have shown significant differences in brain structure, functional connectivity, and long-term outcomes between infants born very preterm and those born moderate- or late-preterm (Dubois et al., 2021, Lemola et al., 2017, Padilla et al., 2015, Romeo et al., 2020).

Another potential source of bias in our study relates to head motion. A recent study (Ryan et al., 2023) has found a small but significant association between gestational age and sleep patterns in preterm infants. Furthermore, growing evidence suggests that head motion during fMRI is not merely a nuisance variable, but is linked to underlying neurophysiological states such as sleep and arousal (Blumberg et al., 2020, Denisova, 2019, Mitra et al., 2017). As a result, conventional motion correction techniques such as frame scrubbing may inadvertently bias the BOLD time series toward specific physiological states, and in this context, toward particular gestational ages. To reduce this risk, we opted to retain a contiguous block of data with the lowest motion, rather than relying on traditional scrubbing approaches.

A final consideration is that all participants were drawn from a single dataset, raising the possibility of residual dependencies at the dataset level (e.g., scanner, acquisition protocol, or population characteristics). While such dependencies are common in empirical-Bayes applications and do not bias estimates within the cohort, they may limit generalizability to other populations. Future studies will extend these analyses across multiple independent datasets to provide a more rigorous test of the robustness and generalizability of the Bayesian framework in larger and more diverse populations.

## 5 Conclusions

In this study, we applied a precision functional mapping approach to characterize individual resting-state network (RSN) patterns in preterm and term-born infants. This individualized method enabled detailed tracking of RSN development from short-duration fMRI scans, preserving subject-specific topography and inter-individual variability. We observed age-related increases in functional connectivity in preterm and term-born infants, particularly within higher-order networks such as the default mode network (DMN), which also demonstrated the most rapid postnatal maturation. Although overall maturation rates were similar across groups, persistent differences in connectivity suggest that RSN disruptions related to prematurity may emerge shortly after birth. These findings highlight the value of subject-level mapping for detecting early developmental alterations and informing future longitudinal studies.

## CRediT authorship contribution statement

**Diego Derman:** Writing – original draft, Writing – review & editing, Visualization, Validation, Software, Resources, Methodology, Investigation, Formal analysis, Data curation, Conceptualization. **Ferradal Silvina L:** Writing – original draft, Writing – review & editing, Supervision, Methodology, Project administration, Funding acquisition, Conceptualization.

## Declaration of Competing Interest

The authors declare that they have no known competing financial interests or personal relationships that could have appeared to influence the work reported in this paper.

## Data Availability

The neonatal data in this study are part of the second release of the developing Human Connectome Project and are available to download (https://www.developingconnectome.org).
